# Fish oil supplement use modifies the relationship between dietary oily fish intake and plasma *n*-3 PUFA levels: an analysis of the UK Biobank

**DOI:** 10.1017/S0007114524000138

**Published:** 2024-05-14

**Authors:** Joanna Aldoori, Michael A. Zulyniak, Giles J. Toogood, Mark A. Hull

**Affiliations:** 1 Leeds Institute of Medical Research, University of Leeds, Leeds LS9 7TF, UK; 2 School of Food Science and Nutrition, University of Leeds, Leeds LS2 9JT, UK; 3 St James’s University Hospital, Leeds Teaching Hospitals NHS Trust, Leeds LS9 7TF, UK

**Keywords:** DHA, Fish oil supplement, *n*-3 PUFA, UK Biobank

## Abstract

Observational evidence linking dietary *n*-3 PUFA intake and health outcomes is limited by a lack of robust validation of dietary intake using blood *n*-3 PUFA levels and potential confounding by fish oil supplement (FOS) use. We investigated the relationship between oily fish intake, FOS use and plasma *n*-3 PUFA levels in 121 650 UK Biobank (UKBB) participants. Ordinal logistic regression models, adjusted for clinical and lifestyle factors, were used to quantify the contribution of dietary oily fish intake and FOS use to plasma *n*-3 PUFA levels (measured by NMR spectroscopy). Oily fish intake and FOS use were reported by 38 % and 31 % of participants, respectively. Increasing oily fish intake was associated with a higher likelihood of FOS use (*P* < 0·001). Oily fish intake ≥ twice a week was the strongest predictor of high total *n*-3 PUFA (OR 6·7 (95 % CI 6·3, 7·1)) and DHA levels (6·6 (6·3, 7·1). FOS use was an independent predictor of high plasma *n*-3 PUFA levels (2·0 (2·0, 2·1)) with a similar OR to that associated with eating oily fish < once a week (1·9 (1·8, 2·0)). FOS use was associated with plasma *n*-3 PUFA levels that were similar to individuals in the next highest oily fish intake category. In conclusion, FOS use is more common in frequent fish consumers and modifies the relationship between oily fish intake and plasma *n*-3 PUFA levels in UKBB participants. If unaccounted for, FOS use may confound the relationship between dietary *n*-3 PUFA intake, blood levels of *n*-3 PUFAs and health outcomes.

The *n*-3 PUFA, EPA and DHA are found in highest quantities in oily fish (such as mackerel, sardines and salmon) and commercially available fish oil supplements (FOS). EPA and DHA are believed to play a beneficial role in the prevention and treatment of several, common, non-communicable diseases including CVD, neurodegenerative diseases, and some cancers, based largely on case–control and cohort epidemiological studies, which have reported the association between oily fish intake, blood *n*-3 PUFA levels and disease outcomes^(1–3)^. However, existing observational evidence linking dietary *n*-3 PUFA intake and health outcomes is often limited to small homogenous studies and is hampered by a lack of robust validation of dietary *n*-3 PUFA intake by blood *n*-3 PUFA levels (usually restricted to a small, nested, case–control validation)^(4,5)^, and the absence of data on FOS use in the majority of studies, despite widespread supplement use^(6–8)^.

The UK Biobank (UKBB) is a well-characterised, diverse, prospective cohort of over a half a million individuals from across the UK, which includes comprehensive dietary data, as well as data on nutritional supplement use^(9)^. In 2021, the UKBB released data on plasma PUFA levels for approximately 120 000 UKBB participants using the Nightingale NMR metabolomics platform^(10)^.

A recent study used UKBB plasma PUFA data to derive an estimated ‘*n*-3 index’ (O3i; the percentage EPA and DHA of total identified PUFAs in erythrocyte membranes) for UKBB participants^(11)^. Schuchardt and colleagues then investigated the relationship between the estimated O3i and several demographic and lifestyle factors, including fish consumption and FOS use, but did not explore the relationship between dietary *n*-3 PUFA intake and FOS use according to the actual plasma *n*-3 PUFA data available in the UKBB.

Herein, we describe the relationship between dietary *n*-3 PUFA intake from oily fish, FOS use and plasma *n*-3 PUFA levels in UKBB participants in order to define the relationship between dietary and supplemental *n*-3 PUFA intake, as well as their relationship with circulating PUFA levels and clinical/lifestyle factors.

## Methods

### The UK Biobank

The UKBB is a large-scale prospective cohort study of 502 441 women and men, aged 40–69 years, recruited between 2006 and 2010^(9)^. Approval for this study was obtained from the UKBB (research ID 73904).

### Assessment of oily fish intake and nutritional supplement use

We examined oily fish intake based on its robust association with increased blood *n*-3 PUFA levels, by contrast with other (lean) fish such as cod, tuna or haddock^(12,13)^.

The self-reported frequency of oily fish intake was assessed using a touchscreen FFQ, reporting typical dietary intake at the time of recruitment^(14)^. We categorised oily fish intake as ‘never’, ‘less than once a week’, ‘once a week’, ‘greater than or equal to twice a week’ (combining UKBB categories ‘2–4 times a week’, ‘5–6 times a week’ and ‘once, or more, daily’) or ‘unknown’ (combining ‘do not know’ and ‘prefer not to answer’ categories), reflecting the distribution of the data and current UK dietary recommendations for oily fish intake (one portion of oily fish per week)^(15)^. Reproducibility of the touchscreen data has been evaluated in a subset of participants (n 320 000), who were invited to complete a 24-h dietary questionnaire on five separate occasions (April 2009 to June 2012)^(16)^.

Nutritional supplement use was examined using the ‘mineral and other dietary supplements use’ question inside the touchscreen FFQ^(17)^. Participants were asked if they regularly used any of the following: ‘fish oil (including cod liver oil)’, ‘glucosamine’, ‘calcium’, ‘iron’, ‘selenium’, ‘none’ and ‘prefer not to answer’. Participants could select more than one answer. We categorised supplement intake as ‘no supplement use’, ‘FOS’, ‘other’ and ‘unknown’ (combining ‘do not know’ and ‘prefer not to answer’ categories). FFQ data were missing for 447 (0·4 %) participants. Nutritional supplement use was also examined using the UKBB 24- dietary recall tool to examine the agreement between the FFQ and 24-h dietary recall for nutritional supplement use^(14)^. Participants were asked ‘did you have any vitamin or mineral supplements yesterday, e.g. vitamin C, multivitamins, fish oil, Ca supplement?’, which was recorded as ‘yes’ or ‘no’. No data were available on the formulation or dose of supplements.

### Plasma fatty acid profiles

A plasma fatty acid profile was available for 121 650 randomly selected UKBB participants using a non-fasted (mean time since last meal 4 h) plasma sample collected in an EDTA-containing tube at either initial UKBB assessment (2006–2010) or the first repeat UKBB assessment (2012–2013), with a plasma fatty acid profile available for both visits in a smaller subset of participants (n 1426)^(10)^.

Plasma fatty acid levels were measured using the Nightingale Health NMR-based metabolic biomarker profiling platform^(10,18)^. The following fatty acid classes were analysed as the absolute plasma concentration in mmol/l: total fatty acids (total FA), total *n*-3 PUFA, total *n*-6 PUFA, DHA and linoleic acid (LA). NMR spectroscopy allows sample analysis at scale, but this methodology is unable to quantify some individual fatty acids including*α*-linolenic acid, arachidonic acid, docosapentaenoic acid and EPA^(18,19)^. The Nightingale fatty acid panel also includes percentage values of total FA for total PUFA, *n*-3 PUFA, *n*-6 PUFA, DHA and LA, in addition to the ratio of total *n*-6 PUFA to total *n*-3 PUFA.

The plasma fatty acid profile for each UKBB participant was linked to touchscreen dietary and nutritional supplement data collected at the same assessment visit.

### Other clinical data

The following variables were also used to describe the population: sex; age (years); BMI (kg/m^2^); ethnicity (White, Mixed, Asian, Black, Chinese, Other and unknown); alcohol intake (never, once to three times a month, once or twice a week, three to four times a week, daily or most days); current tobacco smoking (yes on most or all days, only occasionally, no); physical activity defined using summed metabolic equivalent task (MET) minutes per week for all activities including walking, moderate and vigorous activity (low (< 150 MET × minutes per week), moderate (600 to 3000 MET × minutes per week) and high (> 3000 MET × minutes per week); and Townsend socio-economic deprivation index (quintiles of increasing deprivation (least to most deprived)). Menopausal status (premenopausal, postmenopausal, unsure because of hysterectomy or other reason, unknown), use of hormone replacement therapy (HRT) (use (whether the participant had ever used HRT), no use, unknown) and oral contraceptive (use (whether the participant had ever used oral contraceptive), no use, unknown) were included based on possible sex-specific differences in *n*-3 PUFA levels^(20,21)^.

### Statistical analysis

We examined the complete UKBB population with plasma fatty acid data and accompanying touchscreen data on diet and nutritional supplement use (> 120 000), so statistical justification of the sample size was not necessary. An arbitrary *P* value of < 0·005 was considered statistically significant given the large dataset and propensity for type 1 error due to multiple testing.

Population characteristics were reported as the mean and standard deviation, or as the percentage (%) value. Plasma fatty acid data were log-transformed when appropriate to allow parametric analysis (online Supplementary Fig. 1–3). The *χ* ^2^ test was used to examine the difference between populations with and without plasma fatty acid levels. The Cochrane–Armitage test for trend was used to examine the differences in population characteristics according to oily fish intake and nutritional supplement use. A logistic regression model was used to examine the association between plasma fatty acid levels and oily fish intake. One-way ANOVA with Tukey’s honest significant difference test was used to examine the association between plasma fatty acid levels and nutritional supplement use.

Ordinal logistic regression models were used to analyse multiple factors that predicted the highest quartile of plasma total *n*-3 PUFA levels, plasma DHA levels and the percentage *n*-3 PUFA of total FA. Each model was adjusted for: sex, age at recruitment (≤ 39, 40–49, 50–59 and ≥ 60 years), BMI (≤ 24·9, 25·0–29·9 and ≥ 30 kg/m^2^), oily fish intake frequency (never, < once a week, once a week and ≥ twice a week), supplement use (no supplement use, FOS use and other supplement use), ethnicity (White, Mixed, Asian, Black, Chinese and Other), alcohol intake (never, once to three times a month, once or twice a week, three to four times a week, daily or most days), smoking (non-smoker, occasional smoker and smoker), physical activity (low, moderate or high) and socio-economic deprivation (quintiles of increasing Townsend deprivation index (least to most deprived)).

To examine the modifying effects of menopausal status (premenopausal and postmenopausal), use of HRT (use and no use) and oral contraceptive use (use and no use), the models were re-run for females only. Females who answered ‘unsure – had a hysterectomy’ and ‘unsure-other reason’ within the ‘have you had your menopause?’ question were excluded from the model.

Participants with missing covariate data for the adjusted variables were excluded from the models. Variables within each model were examined for co-linearity. Variables with a moderate or high correlation (based on a variable inflation factor > 5) were removed, and the models were re-derived.

All analyses were performed in R Studio version 4.1.2.

## Results

### Plasma fatty acid profile data

**Fig. 1.**
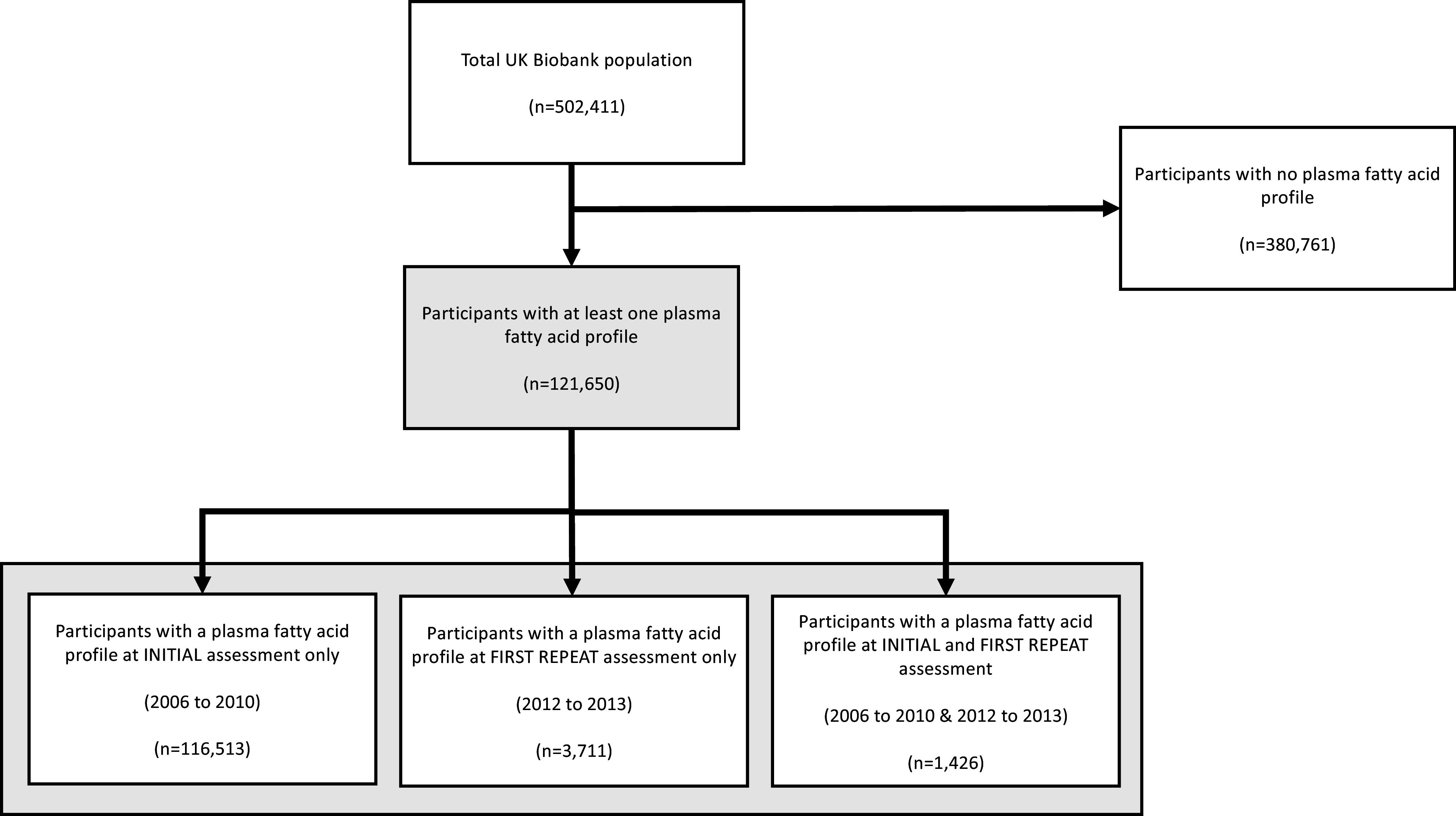
Distribution of plasma fatty acid data in the UK Biobank population. For participants with a plasma fatty acid profile at initial and first repeat assessment, only the initial assessment data were used.

Plasma fatty acid levels were measured at least once in 121 650 participants (24·5 % of the total UKBB population), of which 116 513 had levels measured at the initial assessment visit only, 3711 had plasma fatty acid levels measured at the first repeat assessment visit only and 1426 participants had plasma fatty acid levels measured at both time points (Fig. 1).

A moderate correlation (r^2^ value between 0·52 and 0·57) between initial and repeat assessment plasma fatty acid levels has been reported by the UKBB for a cohort of 1439 participants (our cohort of 1426 likely reflects subsequent participant dropout from the UKBB study)^(10)^. We also examined the absolute difference between paired plasma fatty acid values from initial and repeat blood samples. There was a slightly higher mean value for most fatty acid classes in blood samples from the first repeat UKBB assessment, but the very small absolute differences failed to reach statistical significance, except for plasma total FA (online Supplementary Table 1). Therefore, we used the initial assessment data for individuals who had provided more than one plasma sample (n 1426) to generate a cohort of 121 650 for subsequent analysis (Fig. 1).

Clinical characteristics were compared between the study population with a plasma fatty acid profile (*n* 121 650) and UKBB participants without a plasma fatty acid profile (n 380 761). The groups were well matched for all variables, although some numerically small differences did reach statistical significance given the size of the groups (Table 1). Therefore, we concluded that the population, for which a plasma fatty acid profile was available, was representative of the whole UKBB population.

**Table 1.** Characteristics of UKBB participants with and without a plasma fatty acid profile

	Population with a plasma fatty acid profile		Population with no plasma fatty acid profile		*P*§
Variable	121 650		380 761		
Number of participants	Mean	sd	Mean	sd	
Sex					
Male (%)	55 915	46	173 169	46	0·50
Female (%)	65 735	54	207 592	55	
Age*					
Age at initial assessment (years)	56·5	8·1	56·5	8·1	0·51
BMI*					
BMI at initial assessment (kg/m2)	27·4	4·8	27·4	4·8	0·12
Oily fish intake†					
Never	13 161	11	41 642	11	0·25
< Once a week	39 713	33	125 121	33	0·16
Once a week	46 030	38	142 441	37	0·01
≥ Twice a week	21 886	18	68 337	18	0·73
Unknown	860	1	3220	1	< 0·001
Mineral and dietary supplement use†					
None	69 207	57	213 368	56	< 0·001
Other	13 960	12	43 966	12	0·50
Fish oil	38 036	31	117 680	31	< 0·001
Unknown	447	< 1	5747	1	< 0·001
Menopausal status‡					
Premenopausal	15 423	24	48 623	23	0·83
Postmenopausal	39 781	61	125 599	61	0·95
Not sure as had a hysterectomy or for other reason	10 333	16	32 559	16	0·83
Unknown	198	< 1	811	< 1	< 0·001
Use of hormone replacement therapy‡					
Use	25 026	38	78 879	38	0·73
No use	40 376	61	127 472	61	0·94
Unknown	333	1	1241	1	0·01
Use of oral contraceptive pill‡					
Use	53 036	81	167 356	81	0·72
No use	12 406	19	39 112	19	0·86
Unknown	228	< 1	1124	1	< 0·001
Ethnicity					
White	114 844	94	357 770	94	< 0·001
Non-White	6235	5	20 768	6	< 0·001
Mixed	678	1	2276	1	0·23
Asian	2346	2	7533	2	0·27
Black	1816	2	6242	2	< 0·001
Chinese	353	< 1	1220	< 1	0·10
Other	1060	1	3497	1	0·13
Unknown	553	1	2223	1	< 0·001

UKBB, UK Biobank.

* Mean and standard deviation.

† Oily fish/supplement intake was recorded at the same time as the plasma fatty acid levels were measured. Oily fish intake at initial assessment is reported for the population with no plasma fatty acid profile. Figures in brackets are % values of the whole population.

‡ Percentage (%) of female population.

§ *t* Test for continuous variables and *χ*^2^ test for categorical variables.

### Fish oil supplement use

The 24-h dietary recall data were collected a mean (sd) 2·5 (1·7) years after the corresponding FFQ response. There was a good level of agreement (86 %) between the FFQ and 24-h dietary recall data suggesting that FOS supplement use was consistent over time for the majority of participants (online Supplementary Table 2).

Almost one-third of participants (*n* 38 036, 31 %) used a FOS (online Supplementary Table 3). FOS users had higher oily fish intake than individuals who did not take a supplement (online Supplementary Table 3). This finding was specific for FOS use, as opposed to other supplement use, which is presumably related to shared belief about the health benefits of both dietary and supplemental *n*-3 PUFA intake (online Supplementary Table 3).

Nutritional supplement users were older and more likely to be female than individuals who did not report nutritional supplement use. Nutritional supplement use (including FOS) was common in postmenopausal women, including HRT users. Overall, FOS users were distinct from other nutritional supplement users, who were proportionally more likely to be female, premenopausal, of South Asian ethnicity and report lower frequency of alcohol intake (online Supplementary Table 3).

### Oily fish intake

We also characterised participants according to oily fish intake (online Supplementary Table 4). Dietary intake data were available for 120 790 (99·3 %) participants with a plasma fatty acid profile. The largest proportion of these participants (n 46 030, 38 %) reported eating oily fish once a week, in accordance with UK dietary guidelines^(15)^, with 18 % (n 21 886) reporting consumption of oily fish at least twice a week (online Supplementary Table 4). Participant characteristics that predicted higher oily fish intake were similar to those associated with FOS use, including female sex, postmenopausal status and HRT use (online Supplementary Table 4).

### The relationship between oily fish intake and fish oil supplement use

**Fig. 2.**
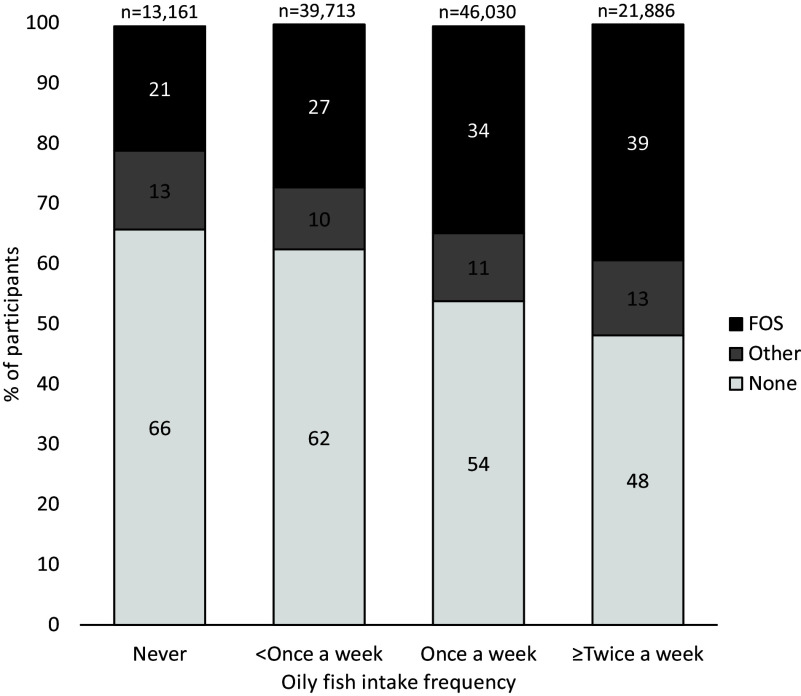
Nutritional supplement use according to dietary oily fish intake frequency of the UKBB study population with a plasma fatty acid profile. See online Supplementary Table 4 for the number of individuals in each supplement use category, including ‘missing data’, which accounted for < 1 % of the study population. FOS, fish oil supplement.

The relationship between dietary oily fish intake and likelihood of FOS use is described in Fig. 2. The relationship between oily fish intake and likelihood of FOS use was evident with a clear ‘dose-dependent’ relationship between increasing frequency of dietary oily fish intake and the proportion taking a FOS, which was not apparent for other supplement use (online Supplementary Table 4). Higher oily fish intake was associated with a larger proportion of FOS users with 8589 (39 %) of individuals, who reported eating oily fish more than or equal to twice a week, using a FOS compared with 21 % of those who reported never eating oily fish (*P* < 0·001) (Fig. 2). By contrast, increasing oily fish intake frequency was not associated with other supplement use (Fig. 2 and online Supplementary Table 4).

### Plasma fatty acid profiles in UK Biobank participants

The distribution of individual plasma fatty acid values in the 121 650 UKBB participants, who were randomly selected for NMR lipidomic profiling, is shown in online Supplementary Fig. 1. Each fatty acid category demonstrated a wide range of values with a positive skew. The distribution of the individual values for the proportion of *n*-3 PUFAs to total FAs was also positively skewed with a corresponding negative skew for the proportion of *n*-6 PUFAs to total FAs (online Supplementary Fig. 2). All plasma fatty acid data were log-transformed prior to analysis of the relationship between plasma fatty acid levels and *n*-3 PUFA intake (online Supplementary Fig. 3).

### The relationship between dietary and supplement *n*-3 PUFA intake and plasma fatty acid levels

Increasing oily fish intake frequency was associated with higher mean plasma total PUFAs, total *n*-3 PUFAs and DHA, with a clear dose–response relationship in each case (Table 2). This was reflected in a stepwise increase in mean total *n*-3 PUFA concentration between 0·07 and 0·1 mmol/l and a stepwise increase in mean DHA level between 0·03 and 0·04 mmol/l across increasing oily fish intake frequency categories (Table 2, online Supplementary Fig. 4). There was a corresponding dose-dependent decrease in plasma total *n*-6 PUFA and LA concentrations associated with increasing oily fish intake which was smaller than that for *n*-3 PUFAs (Table 2). These findings were mirrored by changes in the individual PUFA class proportions reported against total fatty acids (Table 2). There was an approximate 50 % reduction in the *n*-6 PUFA/*n*-3 PUFA ratio in individuals who reported eating oily fish ≥ twice a week compared with those who did not eat oily fish (Table 2, online Supplementary Fig. 4).

**Table 2.** Plasma fatty acid levels reported as absolute concentration or proportion of total fatty acids according to oily fish intake frequency and supplement use

	Oily fish intake category*								Supplement use category†					
	Never (*n* 13 161)		< Once a week (*n* 39 713)		Once a week (*n* 46 030)		≥ Twice a week (*n* 21 886)		No supplement use (*n* 69 207)		Other (*n* 13 960)		Fish oil supplement use (*n* 38 036)	
	Mean	sd	Mean	sd	Mean	sd	Mean	sd	Mean	sd	Mean	sd	Mean	sd
Total fatty acids‡	11·83	2·47	11·86	2·40	11·88	2·38	11·75	2·35	11·77	2·41¶	11·87	2·37**	11·97	2·36††
Total PUFA‡	4·87	0·80	4·93	0·79	5·01	0·80	5·05	0·81	4·92	0·79¶	5·03	0·81**	5·08	0·81††
Total PUFA to total fatty acids§	41·67	3·87	42·08	3·71	42·64	3·68	43·44	3·76	42·25	3·78¶	42·78	3·68**	42·82	3·73
*n*-3 PUFA‡	0·40	0·17	0·47	0·18	0·55	0·21	0·65	0·26	0·49	0·20¶	0·52	0·21**	0·60	0·23††
*n*-3 PUFA to total fatty acids§	3·34	1·09	3·97	1·20	4·61	1·43	5·49	1·87	4·11	1·43¶	4·37	1·52**	4·99	1·64††
DHA‡	0·18	0·06	0·21	0·07	0·24	0·08	0·28	0·10	0·22	0·09¶	0·23	0·08**	0·26	0·09††
DHA to total fatty acids§	1·56	0·47	1·82	0·53	2·09	0·63	2·46	0·81	1·89	0·63¶	2·00	0·66**	2·23	0·71††
*n*-6 PUFA‡	4·47	0·71	4·46	0·67	4·46	0·68	4·41	0·68	4·43	0·68¶	4·51	0·69**	4·48	0·68††
*n*-6 PUFA to total fatty acids§	38·32	3·96	38·12	3·67	38·02	3·55	37·94	3·49	38·14	3·68¶	38·41	3·56**	37·83	3·54††
Linoleic acid‡	3·47	0·71	3·43	0·67	3·42	0·68	3·34	0·70	3·40	0·68¶	3·47	0·70**	3·42	0·69††
Linoleic acid to total fatty acids§	29·55	3·75	29·18	3·39	28·94	3·35	28·56	3·46	29·09	3·44¶	29·42	3·48**	28·73	3·42††
*n*-6 to *n*-3 PUFA^||^	13·02	6·16	10·65	4·27	9·13	3·52	7·76	3·02	10·51	4·64¶	10·03	4·48**	8·45	3·27††

* Participants with oily fish intake data (*n* 120 790). Linear regression model to examine the difference between plasma fatty acid levels and proportions across oily fish intake categories. A statistically significant trend was observed for all plasma PUFA levels and ratios according to increasing oily fish intake frequency categories (*P* < 0·001).

‡ Plasma fatty acid levels are reported as mmol/l, mean and standard deviation.

§ The proportion (as %) of total fatty acids, mean (sd).

|| The ratio of total *n*-6 PUFA to *n*-3 PUFA, mean (sd).

† Participants with mineral and supplement use data (*n* 121 203). ANOVA and Tukey’s test to examine the difference between plasma fatty acid levels and proportions across supplement categories:

¶ *P* < 0·001 for the difference between ‘no supplement use’ and ‘fish oil supplement use’.

** *P* < 0·001 for the difference between ‘no supplement use’ and ‘other’.

†† *P* < 0·001 for the difference between ‘other’ and ‘fish oil supplement use’.

There was significant overlap between individual fatty acid level values in each oily fish intake category, consistent with wide inter-individual variability in PUFA levels related to other genetic and environmental factors governing plasma PUFA levels, independent of dietary PUFA intake (online Supplementary Fig. 4). For example, 2468 (12·7 %) of 21 866 participants who reported eating oily fish ≥ twice a week had a total *n*-3 PUFA level below the median level (0·38 mmol/l) for those who reported never consuming oily fish (online Supplementary Fig. 4).

FOS use was associated with higher plasma concentrations of total PUFA, total *n*-3 PUFA and DHA than for individuals who did not report any supplement use (Table 2). By contrast to the relationship between oily fish intake frequency and PUFA levels, FOS users also demonstrated higher *n*-6 PUFA and LA concentrations than individuals who did not report any supplement use (Table 2). However, this association disappeared when *n*-6 PUFA and LA were recorded as proportions of total FAs (Table 2). Users of other non-FOS supplements had higher *n*-3 PUFA levels than individuals who did not report supplement use, likely due to ‘other supplement’ users having a higher oily fish intake (oily fish ≥ twice a week 20 %) compared with non-supplement users (15 %) (Table 2).

**Fig. 3.**
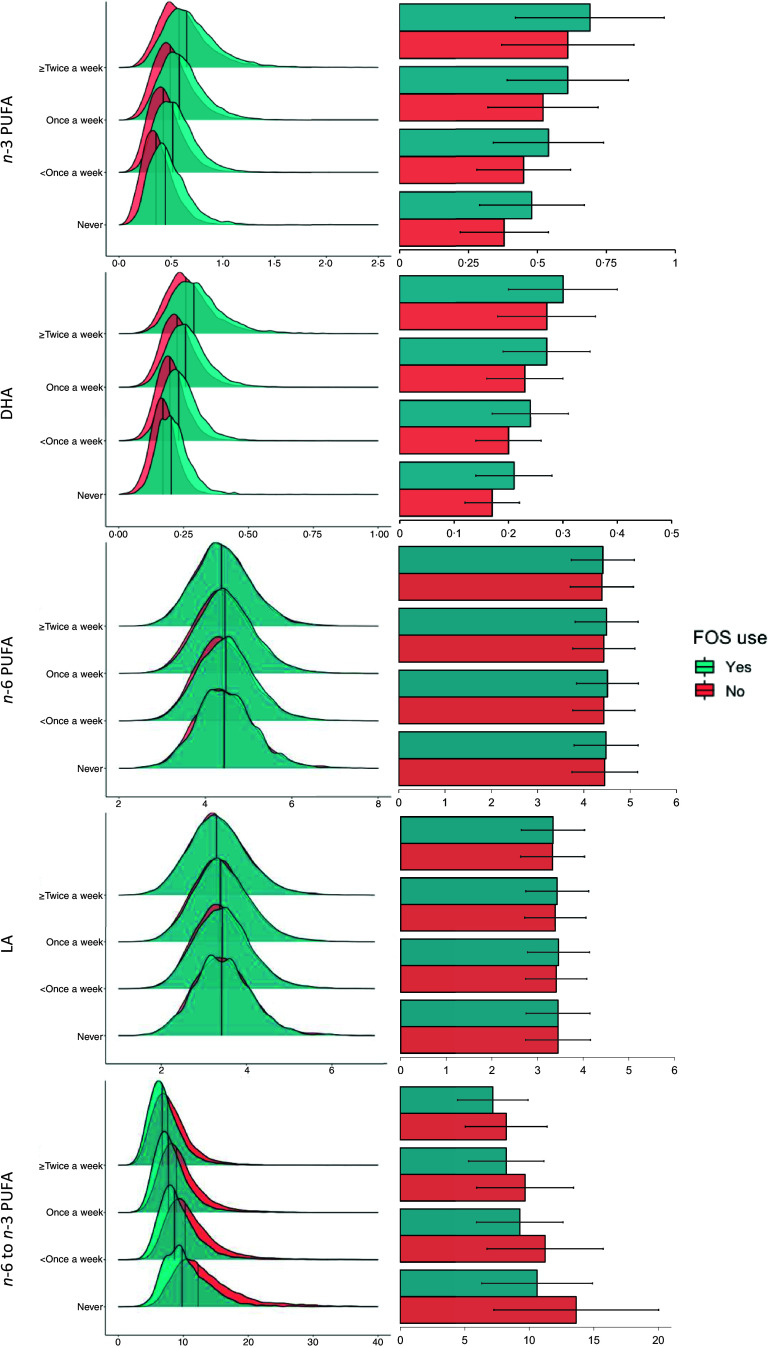
Ridgeline and bar plots showing plasma PUFA levels according to oily fish intake and fish oil supplement use. The X-axis shows the fatty acid concentration in mmol/l, or the *n*-6 to *n*-3 PUFA ratio. Median values are denoted with a dark line in the ridge line plots. Bar plots are the median values with error bars denoting the interquartile range. Green denotes the population who used a fish oil supplement (FOS) compared with the population that did not use a FOS in red. For plasma fatty acid level and ratio values according to oily fish intake and FOS use, see online Supplementary Table 5. LA, linoleic acid.

Given the relationship between oily fish intake and FOS use (Fig. 2), we next examined plasma PUFA levels according to both oily fish intake and FOS use to delineate their respective contributions to plasma PUFA levels (Fig. 3). Comprehensive summary data on fatty acid levels according to both oily fish intake and FOS use are available in online Supplementary Table 5, in which mean and standard deviation values are expressed with their respective median (interquartile range) data. FOS use was consistently associated with an increase in *n*-3 PUFAs, including DHA, and the opposite relationship with the concentration of total *n*-6 PUFAs, including LA (Fig. 3 and online Supplementary Table 5). In general, FOS use was associated with a similar magnitude increase in DHA (+0·3–0·4 mmol/l) and total *n*-3 PUFA level (+0·8–1·0 mmol/l) across all fish intake groups and to a degree whereby the median level of *n*-3 PUFAs of FOS users were comparable to the median *n*-3 PUFA level of an individual in the next highest oily fish intake category who did not consume a FOS (Fig. 3). For example, participants who never ate oily fish but used a FOS had a median (interquartile range) plasma *n*-3 PUFA level of 0·45 (0·35–0·59) mmol/l, while those who ate oily fish less than once a week, but did not use supplements, had a median plasma *n*-3 PUFA level of 0·42 (0·33–0·54) mmol/l (online Supplementary Table 5).

### Factors predicting *n*-3 PUFA levels in UK Biobank participants

We then examined the relationship between plasma *n*-3 PUFA levels and clinical characteristics by building multivariate models that included dietary oily fish intake and supplement use, in addition to other factors that might be associated with plasma *n*-3 PUFA levels. Models were constructed to test the predictive value of these variables for the highest quartile of plasma *n*-3 PUFA and DHA (absolute concentration and as a proportion of total FA) (Table 3)

**Table 3.** Factors predicting plasma *n*-3 PUFA and DHA levels in UKBB participants

Characteristic*	*n*-3 PUFAs†		*n*-3 PUFAs as the proportion of total fatty acids†		DHA†	
	OR	95 % CI	OR	95 % CI	OR	95 % CI
Age						
≤ 49 (reference)	1·0		1·0		1·0	
50–59	1·64	1·58, 1·70	1·38	1·33, 1·43	1·48	1·42, 1·54
> 60	1·92	1·85, 2·00	1·71	1·64, 1·77	1·61	1·55, 1·67
Sex						
Female (reference)	1·0		1·0		1·0	
Male	0·57	0·56, 0·59	0·64	0·62, 0·66	0·38	0·36, 0·39
BMI (kg/m2)						
≤ 24·9 (reference)	1·0		1·0		1·0	
25–29·9	1·29	1·25, 1·33	0·95	0·92, 0·99	0·89	0·86, 0·91
≥ 30	1·20	1·14, 1·23	0·71	0·69, 0·74	0·58	0·55, 0·60
Supplement use						
No supplement use (reference)	1·0		1·0		1·0	
Fish oil use	2·03	1·97, 2·09	2·28	2·21, 2·36	2·16	2·09, 2·23
Oily fish intake						
Never (reference)	1·0		1·0		1·0	
< Once a week	1·93	1·84, 2·03	2·34	2·22, 2·46	2·18	2·07, 2·29
Once a week	3·42	3·25, 3·60	5·02	4·77, 5·29	4·36	4·14, 4·59
≥ Twice a week	6·66	6·29, 7·06	12·07	11·37, 12·82	9·55	9·00, 10·14
Alcohol frequency						
Never (reference)	1·0		1·0		1·0	
Once to three times a month	1·01	0·96, 1·07	0·99	0·94, 1·05	1·06	1·00, 1·12
Once or twice a week	1·17	1·12, 1·23	1·09	1·04, 1·14	1·31	1·25, 1·37
Three to four times a week	1·43	1·40, 1·49	1·31	1·25, 1·37	1·75	1·67, 1·83
Daily or most days	1·64	1·56, 1·72	1·34	1·28, 1·41	2·13	2·03, 2·23
Smoking						
Non-smoker (reference)	1·0		1·0		1·0	
Occasional smoker	0·98	0·90, 1·07	0·82	0·75, 0·89	0·86	0·79, 0·94
Smoker	0·69	0·66, 0·73	0·50	0·47, 0·53	0·54	0·51, 0·57
Ethnicity						
White (reference)	1·0		1·0		1·0	
Mixed	1·20	0·98, 1·47	1·28	1·04, 1·58	1·30	1·06, 1·60
Asian	1·04	0·94, 1·16	1·02	0·92, 1·14	0·92	0·83, 1·02
Black	1·54	1·37, 1·74	4·05	3·56, 4·61	2·40	2·12, 2·71
Chinese	1·34	1·03, 1·75	1·17	0·89, 1·54	1·38	1·03, 1·75
Other	1·48	1·27, 1·72	1·74	1·48, 2·04	1·58	1·35, 1·85
Exercise						
Low (< 600 MET × minutes per week) (reference)	1·0		1·0		1·0	
Moderate (600 to 3000 MET × minutes per week)	1·01	0·95, 1·08	0·98	0·93, 1·05	1·01	0·95, 1·08
High (> 3000 MET × minutes per week)	0·95	0·90, 1·00	0·93	0·88, 0·98	1·01	0·95, 1·07
Deprivation						
Least deprived quintile 1 (–6·26 to −3·96) (reference)	1·0		1·0		1·0	
Quintile 2 (–3·96 to −2·81)	0·96	0·92, 1·00	0·93	0·90, 0·97	0·94	0·90, 0·98
Quintile 3 (–2·81 to −1·36)	0·92	0·89, 0·96	0·91	0·87, 0·95	0·90	0·86, 0·94
Quintile 4 (–1·36 to 1·31)	0·91	0·87, 0·95	0·86	0·82, 0·90	0·89	0·85, 0·93
Most deprived quintile 5 (1·31 to 10·88)	0·82	0·79, 0·86	0·79	0·75, 0·82	0·80	0·76, 0·83

UKBB, UK Biobank; FOS, fish oil supplement.

* Models were adjusted for the following clinical and lifestyle factors: age at recruitment (≤ 39, 40–49, 50–59 and ≥ 60 years); sex (male and female); BMI (≤ 24·9, 25·0–29·9 and ≥ 30 kg/m^2^); supplement use (no supplement use and FOS use); oily fish intake frequency (never, < once a week, once a week and ≥ twice a week); alcohol intake (never, once to three times a month, once or twice a week, three to four times a week, and daily or most days); smoking (non-smoker, occasional smoker and smoker); ethnicity (White, Mixed, Asian, Black, Chinese and Other); exercise, the summed metabolic equivalent task (MET) minutes per week for all activities including walking, moderate and vigorous activity (low, moderate or high); and deprivation (quintiles of increasing Townsend deprivation index (least to most deprived)).

† OR and 95 % CI.

In the multivariate model, FOS use was retained as an independent predictor of higher plasma *n*-3 PUFA levels compared with no supplement use (OR 2·03, 95 % CI (1·97, 2·09)), but the effect size was modest in comparison with the relationship between plasma *n*-3 PUFA levels and oily fish intake (Table 3). The OR for the highest quartile of total plasma *n*-3 PUFAs and DHA in FOS users was similar to the OR associated with oily fish intake less than once weekly (Table 3), consistent with the plasma *n*-3 PUFA concentrations observed in these groups (Fig. 3)

Consistent with existing literature, increasing age, female sex and alcohol intake were predictive of high plasma total *n*-3 PUFAs and DHA levels, whilst there was an inverse relationship with tobacco smoking (Table 3)^(22–24)^. Although excess body weight was positively associated with the plasma total *n*-3 PUFA and DHA concentration, an inverse relationship was observed after correction for total FA concentrations. This may be explained by hypertriacylglycerolaemia (and hence elevated PUFA) associated with obesity^(25–27)^ (Table 3). The removal of covariates (alcohol intake frequency, deprivation, age and exercise) with a variable inflation factor > 5 from the model had no discernible effect on risk estimates (data not shown).

To investigate possible female-specific predictive factors, we generated a model restricted to women only (online Supplementary Table 6). There were no marked differences in OR for individual characteristics compared with the multivariable analysis of the whole study (Table 3). In the female-only model, postmenopausal status was associated with increasing plasma *n*-3 PUFA levels (OR 1·65, 95 % CI (1·53, 1·78)) (online Supplementary Table 6).

## Discussion

We report the largest cohort study of the relationship between dietary oily fish intake, FOS use and plasma PUFA levels, made possible by the release of the first tranche of plasma NMR data on approximately 120 000 UKBB participants.

We highlight that, although oily fish intake is the strongest predictor of plasma *n*-3 PUFA levels, FOS use contributes significantly to the plasma *n*-3 PUFA concentration in UKBB participants. Given that approximately 20–30 % of individuals in UK, Canada, New Zealand and Australia report FOS use^(6–8,28–30)^, we conclude that FOS use could confound the relationship between oily fish intake, plasma *n*-3 PUFA levels and health outcomes, if FOS use is not accounted for. Data unadjusted for FOS use may underlie reports in which the relationship between a disease outcome and dietary *n*-3 PUFA intake is not supported by nested case–control analysis of the same outcome according to blood *n*-3 PUFA levels^(4,5)^.

There is excellent agreement between plasma fatty acid levels measured by the Nightingale Health NMR platform and established GC methods^(10)^. However, there is a paucity of comparable plasma NMR data, with which to draw direct comparisons with other studies. Similar individual plasma PUFA levels have been reported in a large (> one million) cross-sectional study of plasma samples from the USA^(24)^ and a much smaller study of young Canadians^(31)^, which both employed GC for quantification of fatty acid levels. Concerns about the reliability of plasma fatty acid measurements related to fluctuations after eating and recent consumption of alcohol^(32)^ have been allayed by more recent studies demonstrating the reliability of repeated plasma *n*-3 PUFA values over time^(33,34)^.

The literature on population-level circulating fatty acid profiles is also beset by heterogeneity of the blood fraction measured. However, an ancillary study of the 2 × 2 factorial Vitamin D and *n*-3 (VITAL) Trial, which explored the effect of 1 g of mixed *n*-3 PUFA (460 mg EPA and 360 mg DHA) daily and/or vitamin D_3_ (2000 IU) daily *v*. placebo in a healthy US population (n 25 781) on cardiovascular and invasive cancer risk, confirmed excellent agreement between fatty acid levels measured in plasma and erythrocyte membranes^(35)^. We note that plasma *n*-3 PUFA levels are comparable to whole blood anderythrocyte *n*-3 PUFA levels reported in a global survey of 298 studies of healthy adults^(36)^.

Consistent with several previous cohort studies^(5,22,23)^, the strongest predictor of *n*-3 PUFA levels in UKBB participants was oily fish intake, with a clear ‘dose–response’ relationship, which was similar to observations from a double-blind, randomised trial that utilised a capsule intervention approximating to the EPA and DHA content of a portion of oily fish^(37)^, as well as other cross-sectional observational studies^(38,39)^.

Delineation of the separate contributions of FOS use and dietary oily fish intake to circulating *n*-3 PUFA levels in the UKBB has provided unique insight into the relative importance of these sources of *n*-3 PUFAs, with the caveat that the UKBB does not contain data on the dose, formulation or frequency of FOS use. A typical prescription of 1 g of FOS capsule that is available in the UK (Omacor®) provides 0·3 g of EPA + DHA per dose which is equivalent to a weekly dose of 2·1 g (if taken daily). By contrast, one portion of oily fish is estimated to contain 2·8 g of *n*-3 PUFAs. We report plasma *n*-3 PUFA levels for FOS users that are similar to values in UKBB participants who ate oily fish more than once a week, consistent with similar mixed *n*-3 PUFA intake from daily supplement use and marine *n*-3 PUFA exposure from eating one portion of oily fish per week. The UKBB findings suggest a larger effect size of FOS use than a much smaller study of the EPIC Norfolk cohort (*n* 4949), which reported that the effect of FOS use (including cod liver oil (CLO) on plasma *n*-3 PUFA levels was equivalent to eating one-third of a portion of oily fish per week^(40)^.

We appreciate that the relationship between FOS use and plasma *n*-3 PUFA levels may be underestimated by inclusion of CLO use in the UKBB FOS category given the lower content of marine *n*-3 PUFAs in CLO (a 1 g of CLO capsule contains roughly 0·17 g of EPA and DHA combined). Ideally, future studies should include strategies to collect FOS use data that include frequency of use, formulation and dose, in addition to distinguishing FOS from CLO use.

Another limitation of the study is the inability of the NMR technique to distinguish and measure several, key individual PUFAs that are measurable by GC methods, including the *n*-3 PUFA EPA, its metabolite *n*-3 docosapentaenoic acid and its *n*-6 PUFA counterpart arachidonic acid.

That said, the large UKBB dataset of PUFA class values has confirmed the wide variability in plasma fatty acid levels in individuals with a similar *n*-3 PUFA intake^(36,39)^, compatible with strong genetic influences on fatty acid profiles, although the contribution of imprecise reporting of *n*-3 PUFA intake in this and other observational studies should not be underestimated.

It is recognised that several other biological factors are associated with *n*-3 PUFA levels apart from *n*-3 PUFA intake^(41)^. The large UKBB cohort has confirmed findings from other, independent studies that age, female sex and alcohol intake are associated with higher blood *n*-3 PUFA levels^(20,21,24,42)^, with a negative association with BMI^(27)^. Similar conclusions have been reported by Schuchardt and colleagues for the estimated O3i value, which was derived from the original UKBB plasma PUFA data^(11)^.

In conclusion, dietary *n*-3 PUFA intake and FOS use predict plasma *n*-3 PUFA levels in UKBB participants. FOS use was common in UKBB participants and is associated with higher *n*-3 PUFA levels than in those who do not use a FOS. Data on FOS use should be integral to the analysis of all epidemiological data, which examine the link between dietary *n*-3 PUFA intake, blood levels of fatty acids and health outcomes.

## Supporting information

Aldoori et al. supplementary material Aldoori et al. supplementary material

## Supplementary material

For supplementary material/s referred to in this article, please visit https://doi.org/10.1017/S0007114524000138
