# Supplementary material for: Fish oil supplement use modifies the relationship between dietary oily fish intake and plasma n-3 PUFA levels: an analysis of the UK Biobank
Source: Br J Nutr. 2024 Jan 15;131(9):1608–18. doi: 10.1017/S0007114524000138 (PMC11043909; doi:10.1017/S0007114524000138)
Supplement: Aldoori et al. supplementary material [file S0007114524000138sup001.docx]

**Supplementary Materials** **for:**

# Fish oil supplement use modifies the relationship between dietary oily fish intake and plasma omega-3 polyunsaturated fatty acid levels: An analysis of the UK Biobank.

# Aldoori J et al.

**Supplementary Tables**

Supplementary Table 1. Comparison of paired plasma fatty acid data at initial and first repeat assessment visits (n=1,426).

Supplementary Table 2. Agreement between nutritional supplement data from the food frequency questionnaire and corresponding 24-hour dietary recall tool.

Supplementary Table 3. Characteristics of UK Biobank participants with a plasma fatty acid profile according to nutritional supplement use.

Supplementary Table 4. Characteristics of UK Biobank participants with a plasma fatty acid profile according to oily fish intake category.

Supplementary Table 5. Plasma fatty acid classes as the absolute concentration, proportion of total fatty acids, and the omega-6 to omega-3 polyunsaturated fatty acids ratio, according to oily fish intake frequency and supplement use.

Supplementary Table 6. Factors predicting plasma omega-3 polyunsaturated fatty acids and docosahexaenoic acid levels in female UK Biobank participants.

**Supplementary Figures**

Supplementary Figure 1. Distribution of plasma fatty acid levels in the UK Biobank population with a nuclear magnetic resonance plasma fatty acid profile (n=121,650).

Supplementary Figure 2. Distribution of plasma fatty acids as a proportion of total fatty acids and the omega-6 to omega-3 polyunsaturated fatty acid ratio in the UK Biobank population with a nuclear magnetic resonance plasma fatty acid profile (n=121,650).

Supplementary Figure 3. Q-Q plots of log-transformed plasma fatty acid data from the UK Biobank population with a nuclear magnetic resonance plasma fatty acid profile (n=121,650).

Supplementary Figure 4. Ridgeline plots demonstrating the distribution of plasma fatty acid levels and the ratio of omega-6 to omega-3 polyunsaturated fatty acid values in UK Biobank participants with oily fish intake data (n=120,790).

Supplementary Table 1. Comparison of paired plasma fatty acid data at initial and first repeat assessment visits (n=1,426).

|  | **Plasma fatty acid level (mmol/L)^1^** | | **Difference between paired fatty acid values^2^** | **P value^3^** |
| --- | --- | --- | --- | --- |
|  | **Initial assessment** | **First repeat assessment** |  |  |
| **Total fatty acids** | 11.84 (2.39) | 11.95 (2.36) | -0.013 (-0.022 to -0.003) | 0.007 |
| **Total polyunsaturated fatty acids** | 4.98 (0.80) | 5.02 (0.82) | -0.003 (-0.011 to +0.005) | 0.42 |
| **Total omega-3 polyunsaturated fatty acids** | 0.53 (0.22) | 0.54 (0.22) | -0.004 (-0.022 to +0.015) | 0.68 |
| **Docosahexaenoic acid** | 0.23 (0.08) | 0.24 (0.84) | +0.010 (-0.006 to +0.026) | 0.23 |
| **Total omega-6 polyunsaturated fatty acids** | 4.45 (0.68) | 4.48 (0.71) | -0.004 (-0.011 to +0.003) | 0.30 |
| **Linoleic acid** | 3.41 (0.68) | 3.41 (0.71) | +0.006 (-0.004 to +0.016) | 0.23 |
| ^1^ Mean (standard deviation).  **^2^** Mean and 95% confidence interval of the difference between paired initial and repeat assessment plasma fatty acid levels.  ^3^ Paired t-test. | | | | |

Supplementary Table 2. Agreement between nutritional supplement data from the food frequency questionnaire and corresponding 24-hour dietary recall tool.

|  | | **Food frequency questionnaire (FFQ)** | | | |
| --- | --- | --- | --- | --- | --- |
|  |  | **None** | **Other** | **Fish oil supplement use** | **Unknown** |
| **24-hour dietary recall^1^** | **Yes** | 1,145^2^  (12) | 1,380  (66) | 4,445  (86) | 8  (24) |
|  | **No** | 8,566  (88) | 699  (34) | 718  (14) | 26  (77) |
|  | **Total number of participants** | 9,711 | 2,079 | 5,163 | 34 |
| ^1^ Participants were asked “did you use vitamin or mineral supplements yesterday?”  ^2^ Data represent the number of participants with the % of the total in each FFQ supplement category in brackets. | | | | | |

The 24-hour dietary recall question about vitamin and mineral supplement use (collected between April 2009 and September 2010) was compared with the FFQ mineral and supplement use question collected at initial assessment (2006 to 2010). Paired data were available for 16,987 (13.96%) of the population with a plasma fatty acid profile (n=121,650).

There was good agreement between the FFQ fish oil supplement (FOS) use response and 24-hour dietary recall data with 4,445 (86%) individuals, who answered ‘yes’ to FOS use in the FFQ, also answering ‘yes’ to the question about supplement use in the previous 24 hours. Conversely, 88% of individuals who stated no supplement use in the FFQ also answered ‘no’ in the 24-hour recall tool.

Lower agreement between the FFQ and the 24-hour dietary recall tool for other supplement use may reflect less regular use of other non-fish oil supplements, such as glucosamine or zinc.

Supplementary Table 3. Characteristics of UK Biobank participants with a plasma fatty acid profile according to nutritional supplement use.

|  | **No supplement use** | **Other** | **Fish oil supplement use** | **P trend**^3^ |
| --- | --- | --- | --- | --- |
| **Number or participants**  **Percentage of participants (%)** | **69,207**  **(57)** | **13,960**  **(12)** | **38,036**  **(31)** |  |
| **Sex** | | | | |
| Male (%) | 34,651 (50) | 4,344 (31) | 16,675 (44) | <0.001 |
| Female (%) | 34,556 (50) | 9,616 (69) | 21,361 (56) |  |
| **Age^1^** | | | | |
| Age at initial assessment (years) | 55.2 (8.2) | 57.2 (7.8) | 58.7 (7.4) | <0.001 |
| **BMI^1^** | | | | |
| BMI at initial assessment (Kg/m^2^) | 27.6 (4.9)^a^ | 27.2 (4.9)^b^ | 27.2 (4.5) | <0.001 |
| **Oily fish intake** | | | | |
| Never | 8,649 (13) | 1,725 (12) | 2,728 (7) | <0.001 |
| <Once a week | 24,798 (36) | 4,086 (29) | 10,744 (28) |  |
| Once a week | 24,743 (36) | 5,360 (38) | 15,831 (42) |  |
| ≥Twice a week | 10,517 (15) | 2,729 (20) | 8,589 (23) |  |
| Unknown | 500 (1) | 60 (<1) | 144 (<1) |  |
| **Menopausal status^2^** | | | | |
| Pre-menopausal | 10,245 (30) | 1,987 (21) | 3,164 (15) | <0.001 |
| Post-menopausal | 18,750 (54) | 6,121 (64) | 14,846 (70) |  |
| Not sure as had a hysterectomy or for other reason | 5,497 (16) | 1,496 (16) | 3,323 (16) |  |
| Unknown | 64 (<1) | 12 (<1) | 28 (<1) | 0.32 |
| **Use of Hormone replacement therapy^2^** | | | | |
| Use | 11,158 (32) | 3,986 (42) | 9,842 (46) | <0.001 |
| No use | 23,263 (67) | 5,594 (58) | 11,459 (54) |  |
| Unknown | 135 (<1) | 36 (<1) | 60 (<1) | 0.04 |
| **Use of oral contraceptive pill^2^** | | | | |
| Use | 28,408 (82) | 7,678 (80) | 16,884 (79) | <0.001 |
| No use | 6,047 (18) | 1,900 (20) | 4,424 (21) |  |
| Unknown | 101 (<1) | 38 (<1) | 53 (<1) | 0.45 |
| **Ethnicity** | | | | |
| White | 65,368 (95) | 13,037 (93) | 36,207 (95) | <0.001 |
| Non White^3^ | 3,602 (5) | 866 (6) | 1,673 (4) | <0.001 |
| Mixed | 392 (1) | 95 (1) | 187 (1) | 0.18 |
| South Asian | 1,438 (2.) | 345 (3) | 514 (1) | <0.001 |
| Black | 975 (1) | 240 (2) | 567 (2) | 0.18 |
| Chinese | 175 (<1) | 51 (<1) | 123 (<1) | 0.03 |
| Other | 622 (1) | 135 (1) | 282 (1) | 0.01 |
| Unknown | 237 (<1) | 57 (<1) | 156 (<1) | 0.07 |
| **Current tobacco smoking** | | | | |
| No | 49,467 (72) | 10,451 (75) | 28,290 (74) | <0.001 |
| Only occasionally | 1,605 (2) | 266 (2) | 778 (2) | 0.002 |
| Yes, on most or all days | 5,030 (7) | 649 (5) | 1,686 (4) | <0.001 |
| Unknown | 13,105 (19) | 2,594 (19) | 7,282 (19) | 0.48 |
| **Alcohol intake** | | | | |
| Rarely / Never | 10,013 (15) | 2,346 (17) | 5,172 (14) | 0.003 |
| One to three times a month | 6,362 (9) | 1,327 (10) | 3,295 (9) | 0.007 |
| Once or twice a week | 14,475 (21) | 2,792 (20) | 7,920 (21) | 0.54 |
| Three or four times a week | 13,381 (19) | 2,640 (19) | 7,660 (18) | 0.003 |
| Daily or almost daily | 11,860 (17) | 2,265 (16) | 6,704 (19) | 0.09 |
| Unknown | 13,116 (19) | 2,590 (19) | 7,285 (19) | 0.51 |
| **Qualifications** | | | | |
| College or University degree | 23,276 (34) | 4,882 (35) | 11,435 (30) | <0.001 |
| Vocational qualifications | 7,885 (11) | 1,594 (11) | 4,977 (13) | <0.001 |
| Optional national exams at ages 17-18 years | 7,607 (11) | 1,588 (11) | 4,176 (11) | 0.94 |
| National exams at age 16 years | 18,395 (27) | 3,723 (27) | 9,993 (26) | 0.30 |
| None of the above | 11,313 (16) | 2,040 (15) | 7,046 (19) | <0.001 |
| Unknown | 731 (1) | 133 (1) | 409 (1) | 0.87 |
| **Average household income before tax** | | | | |
| <18,000 | 12,998 (19) | 2,674 (19) | 7,845 (21) | <0.001 |
| 18,000 to 30,999 | 14,275 (21) | 3,159 (23) | 9,210 (24) |  |
| 31,000 to 51,999 | 15,727 (23) | 3,111 (22) | 8,144 (21) |  |
| 52,000 to 100,000 | 13,232 (19) | 2,273 (16) | 5,587 (15) |  |
| >100,000 | 3,745 (5) | 558 (4) | 1,306 (3) |  |
| Unknown | 9,230 (13) | 2,185 (16) | 5,944 (16) |  |
| ^1^ Mean with standard deviation shown in brackets  ^2^ Percentage values are for females only  ^3^ Cochran-Armitage test for trend. ^b^ Indicates statistically significant difference between ‘no supplement use’ and ‘fish oil supplement use’. ^b^ Indicates statistically significant difference between ‘no supplement use’ and ‘other’. | | | | |

Supplementary Table 4. Characteristics of UK Biobank participants with a plasma fatty acid profile according to oily fish intake category.

|  | **Never** | **<Once a week** | **Once a week** | **≥Twice a week** | **P value^3^** |
| --- | --- | --- | --- | --- | --- |
| **Number or participants**  **Percentage of participants (%)** | **13,161**  **(11)** | **39,713**  **(33)** | **46,030**  **(38)** | **21,886**  **(18)** |  |
| **Sex** | | | | | |
| Male (%) | 6,310 (48) | 19,039 (48) | 20,339 (44) | 9,767 (45) | <0.001 |
| Female (%) | 6,851 (52) | 20,674 (52) | 25,691 (56) | 12,119 (55) |  |
| **Age^1^** | | | | | |
| Age at initial assessment (years) | 54.1 (8.2) | 55.5 (8.0) | 57.3 (7.9) | 58.3 (7.8) | <0.001 |
| **Body mass index^1^** | | | | | |
| Body mass index at initial assessment (Kg/m^2^) | 27.8 (5.2) | 27.5 (4.8) | 27.3 (4.6) | 27.3 (4.7) | 0.005 |
| **Supplement use** | | | | | |
| No supplement use | 8,649 (66) | 24,798 (62) | 24,743 (54) | 10,517 (48) | <0.001 |
| Other | 1,725 (13) | 4,086 (10) | 5,360 (12) | 2,729 (13) | 0.006 |
| Fish oil | 2,728 (21) | 10,744 (27) | 15,831 (34) | 8,589 (39) | <0.001 |
| Unknown | 59 (<1) | 85 (<1) | 96 (<1) | 51 (<1) | 0.003 |
| **Menopausal status^2^** | | | | | |
| Pre-menopausal | 2,164 (32) | 5,767 (28) | 5,358 (21) | 2,067 (17) | <0.001 |
| Not sure as had a hysterectomy or for other reason | 1,141 (17) | 3,188 (15) | 4,027 (16) | 1,913 (16) | 0.48 |
| Post-menopausal | 3,526 (52) | 11,686 (57) | 16,263 (63) | 8,117 (67) | <0.001 |
| Unknown | 20 (<1) | 33 (<1) | 43 (<1) | 22 (<1) | 0.004 |
| **Use of Hormone replacement therapy^2^** | | | | | |
| Use | 2,235 (33) | 7,150 (35) | 10,271 (40) | 5,263 (43) | <0.001 |
| No use | 4,575 (67) | 13,441 (65) | 15,339 (60) | 6,814 (56) | <0.001 |
| Unknown | 41 (<1) | 83 (<1) | 80 (<1) | 41 (<1) | 0.004 |
| **Use of oral contraceptive pill^2^** | | | | | |
| Use | 5,572 (81) | 17,225(83) | 20,683 (81) | 9,340 (77) | <0.001 |
| No use | 1,247 (18) | 3,394 (16) | 4,927 (19) | 2,741 (23) | <0.001 |
| Unknown | 32 (1) | 55 (<1) | 81 (<1) | 38 (<1) | 0.40 |
| **Current tobacco smoking** | | | | | |
| No | 11,195 (85) | 35,267 (89) | 41,814 (91) | 19,892 (91) | <0.001 |
| Only occasionally | 318 (2) | 1,063 (3) | 1,217 (3) | 581 (3) | 0.38 |
| Yes, on most or all days | 1,639 (13) | 3,362 (9) | 2,974 (7) | 1,402 (6) | <0.001 |
| Unknown | 9 (<1) | 21 (<1) | 25 (<1) | 11 (<1) | 0.60 |
| **Alcohol intake** | | | | | |
| Rarely / Never | 3,965 (30) | 7,365 (19) | 7,651 (17) | 4,146 (19) | <0.001 |
| One to three times a month | 1,749 (13) | 5,012 (13) | 4,681 (10) | 2,154 (10) | <0.001 |
| Once or twice a week | 3,294 (25) | 10,325 (26) | 12,104 (26) | 5,342 (24) | 0.11 |
| Three or four times a week | 2,326 (18) | 8,993 (23) | 11,519 (25) | 5,419 (25) | <0.001 |
| Daily or almost daily | 1,809 (14) | 7,990 (20) | 10,047 (22) | 4,800 (22) | <0.001 |
| Unknown | 18 (<1)) | 28 (<1) | 28 (<1) | 25 (<1) | 0.75 |
| **Ethnicity** | | | | | |
| White | 12,162 (92) | 37,767 (95) | 43,882 (95) | 20,494 (94) | 0.01 |
| Non White^3^ | 953 (7) | 1,816 (5) | 1,922 (4) | 1,282 (6) | 0.0002 |
| Mixed | 77 (1) | 215 (1) | 233 (1) | 148 (1) | <0.001 |
| Asian | 640 (5) | 676 (2) | 619 (1) | 330 (2) | <0.001 |
| Black | 111 (1) | 513 (1) | 521 (1) | 512 (2) | <0.001 |
| Chinese | 21 (<1) | 125 (<1) | 141 (<1) | 58 (<1) | 0.31 |
| Other | 104 (1) | 287 (0.7%) | 408 (1) | 234 (1) | <0.001 |
| Unknown | 46 (<1) | 130 (0.3%) | 226 (<1) | 110 (1) | 0.0002 |
| **Qualifications** | | | | | |
| College or University degree | 3,176 (24) | 13,048 (33) | 15,958 (35) | 7,377 (34) | <0.001 |
| Vocational qualifications | 1,510 (12) | 4,648 (12) | 5,503 (12) | 2,732 (13) | 0.001 |
| Optional national exams at ages 17-18 years | 1,258 (10) | 4,562 (12) | 5,128 (11) | 2,408 (11) | 0.03 |
| National exams at age 16 years | 4,063 (31) | 10,936 (28) | 11,660 (25) | 5,360 (25) | <0.001 |
| None of the above | 2,953 (22) | 6,196 (16) | 7,337 (16) | 3,721 (17) | <0.001 |
| Unknown | 201 (2) | 323 (1) | 444 (1) | 288 (1) | 0.49 |
| **Average household income before tax** | | | | | |
| <18,000 | 3,173 (24) | 7,162 (18) | 8,378 (18) | 4,700 (21) | 0.07 |
| 18,000 to 30,999 | 2,853 (22) | 8,604 (22) | 10,177 (22) | 4,917 (23) | 0.02 |
| 31,000 to 51,999 | 2,851 (22) | 9,512 (24) | 10,156 (22) | 4,418 (20) | <0.001 |
| 52,000 to 100,000 | 1,874 (14) | 7,486 (19) | 8,311 (18) | 3,396 (16) | 0.25 |
| >100,000 | 350 (3) | 1,869 (5) | 2,361 (5) | 1,031 (5) | <0.001 |
| Unknown | 2,060 (16) | 5,080 (13) | 6,647 (14) | 3,424 (16) | <0.001 |
| ^1^ Mean with standard deviation shown in brackets  ^2^ Percentage values are of females only  ^3^ Cochran-Armitage test for trend. | | | | | |

Supplementary Table 5. Plasma fatty acid classes as the absolute concentration, proportion of total fatty acids, and the omega-6 to omega-3 polyunsaturated fatty acids ratio, according to oily fish intake frequency and supplement use.

| **Plasma fatty acid levels**  **(mmol/L)** | | **Never**  **(n=13,161)^1^** | | | **<Once a week**  **(n=39,713)** | | | **Once a week**  **(n=46,030)** | | | **Twice a week**  **(n=21,886)** | | |
| --- | --- | --- | --- | --- | --- | --- | --- | --- | --- | --- | --- | --- | --- |
|  |  | **No supplement use** | **Other** | **Fish oil supplement use** | **No supplement use** | **Other** | **Fish oil supplement use** | **No supplement use** | **Other** | **Fish oil supplement use** | **No supplement use** | **Other** | **Fish oil supplement use** |
| **Number of participants** | | **8,649**  **(65.7%)** | **1,725**  **(13.1%)** | **2,728**  **(20.7%)** | **24,798**  **(62.4%)** | **4,086**  **(10.3%)** | **10,744**  **(27.1%)** | **24,743**  **(53.8%)** | **5,197**  **(11.3%)** | **15,831**  **(34.4%)** | **10,517**  **(48.1%)** | **2,729**  **(12.5%)** | **8,589**  **(39.2%)** |
| Total fatty acids | Mean^2^ | 11.78 (2.50) | 11.84 (2.38) | 11.99 (2.45)^c^ | 11.78 (2.40) | 11.89 (2.44)^b^ | 12.03 (2.35)^c^ | 11.78 (2.39)^a^ | 11.91 (2.33)^b^ | 12.02 (2.36)^c^ | 11.70 (2.38) | 11.80 (2.34) | 11.81 (2.32)^c^ |
|  | Median^3^ | 11.48  (10.04, 13.21) | 11.56  (10.18, 13.20) | 11.77  (10.28, 13.39) | 11.50  (10.11, 13.12) | 11.62  (10.22, 13.23) | 11.78  (10.40, 13.39) | 11.51  (10.12, 13.14) | 11.64  (10.29, 13.27) | 11.77  (10.40, 13.33) | 11.43  (10.07, 13.03) | 11.52  (10.17, 13.15) | 11.57  (10.22, 13.17) |
| Total polyunsaturated fatty acids | Mean | 4.83 (0.80) | 4.95 (0.82)^b^ | 4.96 (0.80)^c^ | 4.88 (0.78)^a^ | 4.97 (0.80)^b^ | 5.05 (0.79)^c^ | 4.95 (0.79)^a^ | 5.06 (0.81) | 5.10 (0.81)^c^ | 5.00 (0.81)^a^ | 5.10 (0.80) | 5.11 (0.82)^c^ |
|  | Median | 4.76  (4.28, 5.30) | 4.88  (4.39, 5.42) | 4.90  (4.42, 5.46) | 4.82  (4.35, 5.35) | 4.93  (4.42, 5.44) | 5.01  (4.51, 5.53) | 4.90  (4.40, 5.44) | 5.00  (4.50, 5.56) | 5.05  (4.55, 5.58) | 4.95  (4.45, 5.49) | 5.04  (4.53, 5.61) | 5.06  (4.55, 5.62) |
| Omega-3 polyunsaturated fatty acids | Mean | 0.38 (0.16) | 0.28 (0.15)^b^ | 0.48 (0.19)^c^ | 0.45 (0.17)^a^ | 0.47 (0.18)^b^ | 0.54 (0.20)^c^ | 0.52 (0.20)^a^ | 0.54 (0.20)^b^ | 0.61 (0.22)^c^ | 0.61 (0.24)^a^ | 0.64 (0.24)^b^ | 0.69 (0.27)^c^ |
|  | Median | 0.36  (0.27, 0.46) | 0.36  (0.28, 0.47) | 0.45  (0.35, 0.59) | 0.42  (0.33, 0.54) | 0.45  (0.34, 0.57) | 0.52  (0.40, 0.65) | 0.49  (0.38, 0.62) | 0.52  (0.41, 0.65) | 0.58  (0.46, 0.73) | 0.57  (0.44, 0.73) | 0.61  (0.47, 0.77) | 0.65  (0.51, 0.83) |
| Docosahexaenoic acid | Mean | 0.17 (0.05)^a^ | 0.18 (0.05)^b^ | 0.21 (0.07)^c^ | 0.20 (0.06)^a^ | 0.21 (0.06)^b^ | 0.24 (0.07)^c^ | 0.23 (0.07)^a^ | 0.24 (0.08)^b^ | 0.27 (0.08)^c^ | 0.27 (0.09)^a^ | 0.28 (0.09)^b^ | 0.30 (0.10)^c^ |
|  | Median | 0.17  (0.14, 0.20) | 0.17  (0.15, 0.21) | 0.20  (0.17, 0.24) | 0.20  (0.16, 0.23) | 0.20  (0.17, 0.25) | 0.23  (0.19, 0.28) | 0.22  (0.18, 0.27) | 0.23  (0.19, 0.28) | 0.26  (0.21, 0.31) | 0.25  (0.21, 0.32) | 0.27  (0.22, 0.34) | 0.29  (0.23, 0.36) |
| Omega-6 polyunsaturated fatty acids | Mean | 4.45 (0.71)^a^ | 4.56 (0.73)^b^ | 4.48 (0.69) | 4.43 (0.67)^a^ | 4.50 (0.69) | 4.51 (0.67)^c^ | 4.43 (0.67)^a^ | 4.51 (0.69) | 4.49 (0.68)^c^ | 4.39 (0.68)^a^ | 4.46 (0.67) | 4.41 (0.68) |
|  | Median | 4.40  (3.97, 4.87) | 4.51  (4.07, 5.00) | 4.44  (4.02, 4.89) | 4.39  (4.00, 4.84) | 4.46  (4.03, 4.92) | 4.48  (4.04, 4.92) | 4.39  (3.97, 4.85) | 4.47  (4.05, 4.94) | 4.45  (4.03, 4.90) | 4.35  (3.93,4.80) | 4.42  (3.99, 4.89) | 4.38  (3.97, 4.83) |
| Linoleic acid | Mean | 3.45 (0.71)^a^ | 3.58 (0.75)^b^ | 3.45 (0.70) | 3.41 (0.67)^a^ | 3.47 (0.69) | 3.46 (0.68)^c^ | 3.39 (0.67)^a^ | 3.47 (0.69)^b^ | 3.43 (0.69)^c^ | 3.33 (0.70)^a^ | 3.40 (0.69)^b^ | 3.34 (0.69) |
|  | Median | 3.40  (2.97, 3.87) | 3.51  (3.08, 4.01) | 3.41  (2.99, 3.86) | 3.37  (3.00, 3.82) | 3.42  (3.00, 3.87) | 3.43  (3.00, 3.87) | 3.35  (2.93, 3.80) | 3.42  (3.01, 3.90) | 3.38  (2.97, 3.85) | 3.28  (2.85, 3.75) | 3.35  (2.92, 3.83) | 3.30  (2.87, 3.76) |
| Omega-6 to Omega-3 polyunsaturated fatty acids ratio | Mean | 13.63 (6.38) | 13.86 (6.63)^b^ | 10.60 (4.29)^c^ | 11.22 (4.50)^a^ | 10.83 (4.17)^b^ | 9.26 (3.35)^c^ | 9.67 (3.75)^a^ | 9.29 (3.43)^b^ | 8.21 (2.92)^c^ | 8.21 (3.17)^a^ | 7.84 (2.97)^b^ | 7.17 (2.73)^c^ |
|  | Median | 12.23 (9.86,15.69) | 12.43  (9.90, 15.71) | 9.79  (7.92, 12.30) | 10.34  (8.49, 12.90) | 9.98  (8.13, 12.44) | 8.63  (7.15, 10.70) | 8.98  (7.35, 11.15) | 8.69  (7.08, 10.66) | 7.70  (6.32, 9.48) | 7.69  (6.13, 9.65) | 7.34  (5.92, 9.17) | 6.73  (5.43, 8.42) |

| **Percentage of total fatty acids (%)** | | **Never**  **(n=13,161)^1^** | | | **<Once a week**  **(n=39,713)** | | | **Once a week**  **(n=46,030)** | | | **Twice a week**  **(n=21,886)** | | |
| --- | --- | --- | --- | --- | --- | --- | --- | --- | --- | --- | --- | --- | --- |
|  |  | **No supplement use** | **Other** | **Fish oil supplement use** | **No supplement use** | **Other** | **Fish oil supplement use** | **No supplement use** | **Other** | **Fish oil supplement use** | **No supplement use** | **Other** | **Fish oil supplement use** |
| **Number of participants** | | **8,649**  **(65.7%)** | **1,725**  **(13.1%)** | **2,728**  **(20.7%)** | **24,798**  **(62.4%)** | **4,086**  **(10.3%)** | **10,744**  **(27.1%)** | **24,743**  **(53.8%)** | **5,197**  **(11.3%)** | **15,831**  **(34.4%)** | **10,517**  **(48.1%)** | **2,729**  **(12.5%)** | **8,589**  **(39.2%)** |
| Polyunsaturated fatty acids | Mean | 41.50 (3.90)^a^ | 42.25 (3.82)^b^ | 41.82 (3.75)^c^ | 41.92 (3.73)^a^ | 42.28 (3.66) | 42.40 (3.65)^c^ | 42.45 (3.69)^a^ | 42.89 (3.56) | 42.84 (3.69)^c^ | 43.19 (3.78)^a^ | 43.66 (3.71) | 43.66 (3.75)^c^ |
|  | Median | 42.02  (39.16, 44.26) | 42.69  (40.15, 44.84) | 42.26  (39.47, 44.52) | 42.25  (39.69, 44.59) | 42.83  (40.23, 44.78) | 42.95  (40.26, 45.00) | 42.98  (40.33, 45.04) | 43.42  (40.98, 45.34) | 43.38  (40.72, 45.45) | 43.64  (40.99, 45.78) | 44.02  (41.58, 46.21) | 44.14  (41.49, 46.26) |
| Omega-3 polyunsaturated fatty acids | Mean | 3.17 (0.98) | 3.20 (0.99)^b^ | 2.97 (1.24)^c^ | 3.75 (1.10)^a^ | 3.91 (1.13)^b^ | 4.48 (1.30)^c^ | 4.35 (1.33)^a^ | 4.55 (1.37)^b^ | 5.05 (1.49)^c^ | 5.20 (1.78)^a^ | 5.44 (1.80)^b^ | 5.87 (1.93)^c^ |
|  | Median | 3.12  (2.51, 3.78) | 3.16  (2.55, 3.79) | 3.87  (3.15, 4.64) | 3.68  (3.01, 4.38) | 3.82  (3.14, 4.58) | 4.37  (3.61, 5.19) | 4.22  (3.46, 5.08) | 4.41  (3.64, 5.32) | 4.90  (4.03, 5.85) | 4.93  (3.98, 6.09) | 5.20  (4.20, 6.41) | 5.63  (4.54, 6.83) |
| Docosahexaenoic acid | Mean | 1.49 (0.43) | 1.53 (0.43)^b^ | 1.79 (0.53)^c^ | 1.74 (0.50)^a^ | 1.81 (0.51)^b^ | 2.01 (0.57)^c^ | 1.99 (0.59)^a^ | 2.08 (0.60)^b^ | 2.25 (0.65)^c^ | 2.35 (0.77)^a^ | 2.45 (0.78)^b^ | 2.60 (0.83)^c^ |
|  | Median | 1.49  (1.22, 1.77) | 1.54  (1.25, 1.79) | 1.76  (1.45, 2.07) | 1.72  (1.42, 2.03) | 1.79  (1.49, 2.11) | 1.97  (1.64, 2.34) | 1.95  (1.60, 2.33) | 2.03  (1.69, 2.42) | 2.20  (1.82, 2.61) | 2.24  (1.82, 2.75) | 2.36  (1.92, 2.88) | 2.52  (2.05, 3.05) |
| Omega-6 polyunsaturated fatty acids | Mean | 38.33 (3.99)^a^ | 39.04 (3.95)^b^ | 37.85 (3.80)^c^ | 38.17 (3.71)^a^ | 38.37 (3.64)^b^ | 37.92 (3.59)^c^ | 38.10 (3.58)^a^ | 38.34 (3.43)^b^ | 37.79 (3.51)^c^ | 37.99 (3.54) | 38.22 (3.40)^b^ | 37.79 (3.45)^c^ |
|  | Median | 38.82  (35.90, 41.17) | 39.54  (36.64, 41.85) | 38.32  (35.39, 40.59) | 38.69  (35.90, 40.85) | 38.91  (36.25, 40.97) | 38.49  (35.82, 40.44) | 38.61  (36.01, 40.65) | 38.87  (36.39, 40.76) | 38.29  (35.77, 40.27) | 38.47  (35.92, 40.49) | 38.63  (36.31, 40.54) | 38.24  (35.79, 40.23) |
| Linoleic acid | Mean | 29.56 (3.73)^a^ | 30.43 (3.92)^b^ | 29.00 (3.58)^c^ | 29.22 (3.38)^a^ | 29.44 (3.44)^b^ | 28.98 (3.38)^c^ | 29.00 (3.33)^a^ | 29.32 (3.32)^b^ | 28.71 (3.37)^c^ | 28.61 (3.46)^a^ | 28.94 (3.42)^b^ | 28.38 (3.47)^c^ |
|  | Median | 29.73  (27.18, 31.97) | 30.60  (27.94, 33.15) | 29.15  (26.69, 31.43) | 29.46  (27.10, 31.54) | 29.66  (27.28, 31.80) | 29.19  (26.86, 31.30) | 29.23  (26.91, 31.29) | 29.49  (27.25, 31.57) | 28.93  (26.62, 31.02) | 28.78  (26.42, 30.98) | 29.09  (26.69, 31.29) | 28.51  (26.22, 30.75) |
| ^1^ Missing data in each oily fish category: Never (n=59) 0.4%, <Once a week (n=85) 0.2%, Once a week (n=259) 0.6%, ≥Twice a week (n=51) 0.2%.  ^2^ Mean (standard deviation).  ^3^ Median (interquartile range).  ANOVA and Tukey’s test to examine the difference between plasma fatty acid levels and proportions across supplement categories within oily fish intake frequency categories. ^a^ P<0.005 for the difference between ‘no supplement use’ and ‘fish oil supplement use’,  ^b^ P<0.005 for the difference between ‘no supplement use’ and ‘other’, and ^c^ P<0.005 for the difference between ‘other’ and ‘fish oil supplement use’. | | | | | | | | | | | | | |

Supplementary Table 6. Factors predicting plasma omega-3 polyunsaturated fatty acids and docosahexaenoic acid levels in female UK Biobank participants^[[1]](#footnote-1)^.

| **Characteristics^1^** | **Quartiles of omega-3 polyunsaturated fatty acids^2^** | **Quartiles of omega-3 polyunsaturated fatty acids to total fatty acids ratio^2^** | **Quartiles of docosahexaenoic acid^3^** |
| --- | --- | --- | --- |
| **Age** | | | |
| ≤49 | 1.0 | 1.0 | 1.0 |
| 50-59 | 1.59 (1.47 – 1.72) | 1.30 (1.20 – 1.40) | 1.39 (1.29 – 1.50) |
| >60 | 2.11 (1.93 – 2.31) | 1.65 (1.51 – 1.81) | 1.68 (1.53 – 1.83) |
| **BMI (kg/m^2^)** | | | |
| ≤24.9 (reference) | 1.0 | 1.0 | 1.0 |
| 25-29.9 | 1.20 (1.15– 1.26) | 0.91 (0.87 – 0.96) | 0.86 (0.82 – 0.90) |
| ≥30 | 1.04 (0.99 – 1.11) | 0.64 (0.60 – 0.67) | 0.54 (0.51 – 0.57) |
| **Supplement use** | | | |
| No supplement use (reference) | 1.0 | 1.0 | 1.0 |
| Fish oil use | 2.07 (1.97 – 2.17) | 2.33 (2.22 – 2.44) | 2.24 (2.13 – 2.35) |
| **Oily fish intake** | | | |
| Never (reference) | 1.0 | 1.0 | 1.0 |
| <Once a week | 2.08 (1.92 – 2.25) | 2.59 (2.39 – 2.80) | 2.38 (2.20 – 2.57) |
| Once a week | 3.76 (3.48 – 4.06) | 5.65 (5.22 – 6.11) | 4.79 (4.43 – 5.18) |
| ≥Twice a week | 7.06 (6.46 – 7.71) | 12.76 (11.64 – 13.97) | 9.83 (8.98 – 10.75) |
| **Use of oral contraceptive pill** | | | |
| No use (reference) | 1.0 | 1.0 | 1.0 |
| Use | 0.99 (0.93 – 1.05) | 0.97 (0.91 – 1.03) | 1.02 (0.96 – 1.08) |
| **Use of hormone replacement therapy** | | | |
| No use (reference) | 1.0 | 1.0 | 1.0 |
| Use | 1.03 (0.98 – 1.09) | 1.05 (0.99 – 1.10) | 1.04 (0.99 – 1.09) |
| **Had menopause** | | | |
| No (reference) | 1.0 | 1.0 | 1.0 |
| Yes | 1.65 (1.53 – 1.78) | 1.27 (1.17 – 1.37) | 1.46 (1.35 – 1.58) |
| **Alcohol frequency** | | | |
| Never (reference) | 1.0 | 1.0 | 1.0 |
| Once to three times a month | 1.00 (0.93 – 1.08) | 1.00 (0.92 – 1.07) | 1.08 (1.00 – 1.16) |
| Once or twice a week | 1.17 (1.10 – 1.24) | 1.12 (1.05 – 1.19) | 1.36 (1.28 – 1.45) |
| Three to four times a week | 1.41 (1.32 – 1.51) | 1.34 (1.26 – 1.44) | 1.78 (1.67 – 1.91) |
| Daily or most days | 1.56 (1.46 – 1.68) | 1.34 (1.25 – 1.44) | 2.11 (1.97 – 2.27) |
| **Smoking** | | | |
| Non-smoker (reference) | 1.0 | 1.0 | 1.0 |
| Occasional smoker | 0.84 (0.72 – 0.97) | 0.72 (0.62 – 0.84) | 0.75 (0.64 – 0.86) |
| Smoker | 0.70 (0.64 – 0.76) | 0.49 (0.45 – 0.54) | 0.53 (0.49 – 0.58) |
| **Ethnicity** | | | |
| White (reference) | 1.0 | 1.0 | 1.0 |
| Mixed | 1.24 (0.93 – 1.66) | 1.26 (0.95 – 1.68) | 1.25 (0.94 – 1.67) |
| Asian | 0.90 (0.76 – 1.07) | 0.86 (0.73 – 1.03) | 0.80 (0.68 – 0.95) |
| Black | 1.24 (1.04 – 1.47) | 3.07 (2.55 – 3.69) | 1.79 (1.50 – 2.13) |
| Chinese | 1.92 (1.32 – 2.79) | 1.66 (1.12 – 2.44) | 1.85 (1.26 – 2.71) |
| Other | 1.43 (1.15 – 1.78) | 1.64 (1.30 – 2.06) | 1.53 (1.22 – 1.91) |
| **Exercise ^2^** | | | |
| Low (<600 MET x minutes per week) | 1.0 | 1.0 | 1.0 |
| Moderate (600 to 3,000 MET x minutes per week) | 0.99 (0.90 – 1.09) | 0.92 (0.84 – 1.02) | 0.99 (0.90 – 1.09) |
| High (>3000 MET x minutes per week) | 0.96 (0.88 – 1.05) | 0.88 (0.81 – 0.97) | 0.99 (0.91 – 1.08) |
| **Deprivation^3^** | | | |
| Least deprived Quintile 1 (-6.26 to -3.96) | 1.0 | 1.0 | 1.0 |
| Quintile 2 (-3.96 to -2.81) | 0.99 (0.93 – 1.06) | 0.97 (0.91 – 1.04) | 0.99 (0.93 – 1.06) |
| Quintile 3 (-2.81 to -1.36) | 0.95 (0.89 – 1.02) | 0.93 (0.87 – 1.00) | 0.93 (0.87 – 0.99) |
| Quintile 4 (-1.36 to 1.31) | 0.93 (0.87 – 1.00) | 0.88 (0.82 – 0.94) | 0.92 (0.86 – 0.98) |
| Most deprived Quintile 5 (1.31 to 10.88) | 0.85 (0.80 – 0.91) | 0.80 (0.74 – 0.86) | 0.82 (0.77 – 0.88) |
| **^1^** Models were adjusted for the following clinical and lifestyle factors: age at recruitment (≤39, 40-49, 50-59, ≥60 years); sex (male, female); BMI (≤24.9, 25.0 to 29.9, ≥30 Kg/m^2^); supplement use (no supplement use, FOS use); oily fish intake frequency (never, <once a week, once a week, ≥twice a week); use of oral contraceptive pill (no use, use); use of hormone replacement therapy (no use, use); had menopausal status (no, yes); alcohol intake (never, once to three times a month, once or twice a week, three to four times a week, daily or most days); smoking (non-smoker, occasional smoker, smoker); ethnicity (White, Mixed, Asian, Black, Chinese and Other); exercise, the summed metabolic equivalent task (MET) minutes per week for all activities including walking, moderate and vigorous activity (low, moderate, or high); and deprivation (quintiles of increasing Townsend deprivation index [least to most deprived]).  **^2^** Odd ratio and 95% confidence intervals. | | | |

Supplementary Figure 1. Distribution of plasma fatty acid levels in the UK Biobank population with a nuclear magnetic resonance plasma fatty acid profile (n=121,650).

The X axis for each panel is the fatty acid concentration in mmol/L. The mean plasma fatty acid value for each fatty acid class or individual polyunsaturated fatty acid (PUFA) is denoted by a red line. The median plasma fatty acid value for each class or individual PUFA is denoted by a green line. In each case, the inset panel is the corresponding Q-Q plot.

Supplementary Figure 2. Distribution of plasma fatty acids as a proportion of total fatty acids and the omega-6 to omega-3 polyunsaturated fatty acid ratio in the UK Biobank population with a nuclear magnetic resonance plasma fatty acid profile (n=121,650).

The mean plasma fatty acid proportion (%) or ratio value is denoted by a red line. The median plasma fatty acid proportion or ratio value is denoted by a green line. In each case the inset panel is the corresponding Q-Q plot.

Supplementary Figure 3. Q-Q plots of log-transformed plasma fatty acid data from the UK Biobank population with a nuclear magnetic resonance plasma fatty acid profile (n=121,650).

Supplementary Figure 4. Ridgeline plots demonstrating the distribution of plasma fatty acid levels and the ratio of omega-6 to omega-3 polyunsaturated fatty acid values in UK Biobank participants with oily fish intake data (n=120,790).

The median value for plasma fatty acid concentration (mmol/L) and ratio of omega-6 PUFAs to omega-3 polyunsaturated fatty acids is denoted by the dark vertical line.

1. Model adjusted for female sex and characteristics shown, missing data removed from the model (n=42,704). [↑](#footnote-ref-1)
